# Reconstruction of Partial Achilles Tendon Rupture After Corticosteroid Injection Using the Midsubstance SpeedBridge Technique With Platelet-Rich Plasma: A Case Report

**DOI:** 10.7759/cureus.104965

**Published:** 2026-03-10

**Authors:** Kazuyoshi Minamino, Takahide Sasaki, Tomohiro Matsui, Yukihiro Nakagawa, Hiroshi Yamada

**Affiliations:** 1 Orthopedic Surgery, Saiseikai Wakayama Hospital, Wakayama, JPN; 2 Orthopedic Surgery, Wakayama Medical University Kihoku Hospital, Wakayama, JPN; 3 Sports Medicine, Orthopedic Foot and Ankle Center, Takanohara Central Hospital, Nara, JPN; 4 Orthopedic Surgery, Wakayama Medical University, Wakayama, JPN

**Keywords:** achilles midsubstance speedbridge technique, achilles tendon rupture, corticosteroid injection, minimally invasive surgery, platelet rich plasma injection

## Abstract

In cases of Achilles tendon rupture following local corticosteroid injection, simple end-to-end repair is often technically difficult because of tendon degeneration, and more invasive Achilles tendon reconstruction is commonly required. We report the case of a 56-year-old man with a partial Achilles tendon rupture after local corticosteroid injection who was treated using the Midsubstance SpeedBridge technique combined with platelet-rich plasma (PRP) injection and achieved a favorable clinical outcome. This case suggests that a minimally invasive strategy combining the Midsubstance SpeedBridge technique with biologic therapy may be a feasible therapeutic option for Achilles tendon rupture associated with prior local corticosteroid injection.

## Introduction

Steroids inhibit collagen synthesis, suppress tenocyte proliferation, and reduce local blood flow, thereby promoting tendon degeneration and decreasing mechanical strength [1]. Local corticosteroid injection around the Achilles tendon has been associated with an increased risk of tendon rupture, and several cases of rupture following such injections have been reported [2,3]. In patients who sustain Achilles tendon rupture after local corticosteroid injection, simple end-to-end repair is often technically difficult because of tendon degeneration and structural fragility. Consequently, reconstructive procedures, including tendon transfer and autograft, are frequently required [2,4].

Partial Achilles tendon rupture is defined as a localized discontinuity within the tendon substance [5]. Approximately 50% of affected patients have a history of local corticosteroid injection [6]. However, a clear diagnostic and therapeutic consensus for this condition has not yet been established [5]. Surgical management is typically determined by the extent of the rupture and may involve lesion excision with or without suture adaptation, tendon augmentation, or tendon transfer reconstruction [5]. Extensive partial ruptures, particularly those occurring after corticosteroid injection, often necessitate reconstructive procedures. Nevertheless, such procedures are invasive and are associated with an increased risk of complications, including delayed wound healing and postoperative infection [7,8].

In recent years, the Achilles Midsubstance SpeedBridge technique has been introduced as a minimally invasive procedure using suture anchors to provide strong internal fixation for Achilles tendon rupture, with favorable clinical outcomes reported in the literature [9,10]. Platelet-rich plasma (PRP), which is rich in platelet-derived growth factors, has also been recognized as a biologic therapy with the potential to promote healing of injured Achilles tendon tissue [11]. To the best of current knowledge, no previous report has described the use of the Achilles Midsubstance SpeedBridge technique combined with PRP injection for the treatment of partial Achilles tendon rupture following local corticosteroid injection. This report describes a case of an extensive partial Achilles tendon rupture after local corticosteroid injection that was treated with minimally invasive Achilles tendon reconstruction using the Achilles Midsubstance SpeedBridge technique combined with PRP injection.

## Case presentation

A 56-year-old non-smoking man presented with a several-year history of pain in the right Achilles tendon region. He had previously been diagnosed with insertional Achilles tendinopathy at another institution and had received a total of three local corticosteroid injections into the right Achilles tendon insertion over the preceding five years. One month after the final injection, swelling of the right Achilles tendon region developed in the absence of an apparent traumatic event. The patient was referred to our department six months later for further diagnostic evaluation and treatment. He was 163.0 cm in height and weighed 60.4 kg, corresponding to a body mass index of 22.7 kg/m². His medical history was significant for hypertension, and he had no history of hyperlipidemia or diabetes mellitus. He had not received systemic corticosteroid therapy and had no history of treatment with fluoroquinolone antibiotics. His regular sports activity was recreational golf.

**Figure 1:**
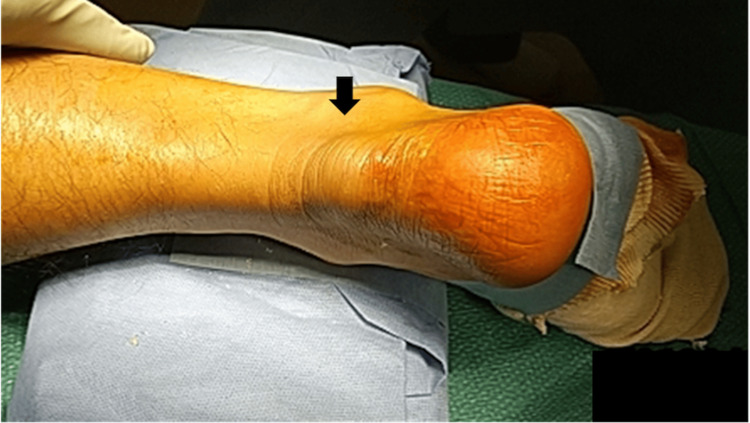
Clinical photograph of the Achilles tendon region The photograph was taken immediately before surgery, not at the first visit. A soft, elastic mass measuring approximately 3 cm was observed at the distal portion of the Achilles tendon (arrow).

At the initial visit to our hospital, physical examination revealed an approximately 3-cm, soft, elastic mass in the distal portion of the right Achilles tendon (Figure 1; the photograph was taken immediately before surgery, not at the first visit). Palpation demonstrated preserved continuity of the tendon, although the tissue was fragile. The Thompson test was negative; however, the plantar flexion response was clearly reduced compared with the contralateral side. Ankle plantar flexion strength was decreased, and the patient was unable to perform a single-leg heel raise on the affected side. The American Orthopaedic Foot and Ankle Society (AOFAS) Ankle-Hindfoot scale at presentation was 68 points. The AOFAS Ankle-Hindfoot scale was developed by the AOFAS and is free to use [12].

**Figure 2:**
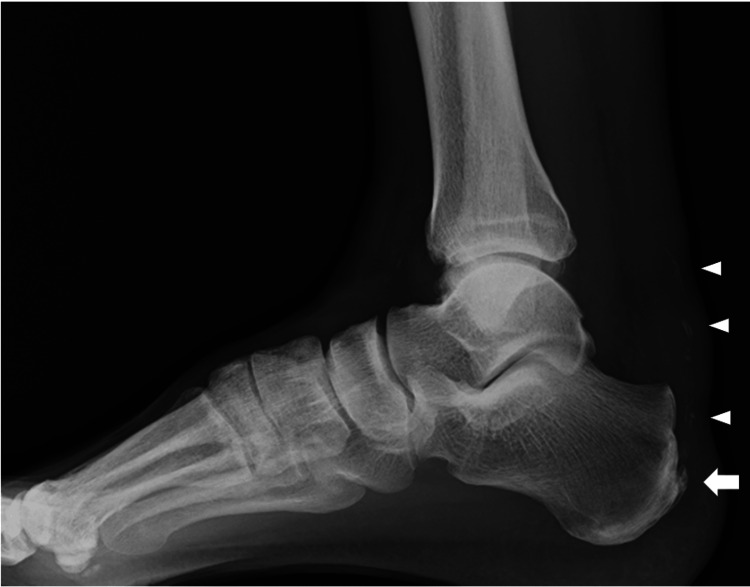
Preoperative plain radiograph A bone spur was present at the calcaneal insertion of the Achilles tendon (arrow), and a calcified lesion was identified along the course of the tendon (arrowheads).

**Figure 3:**
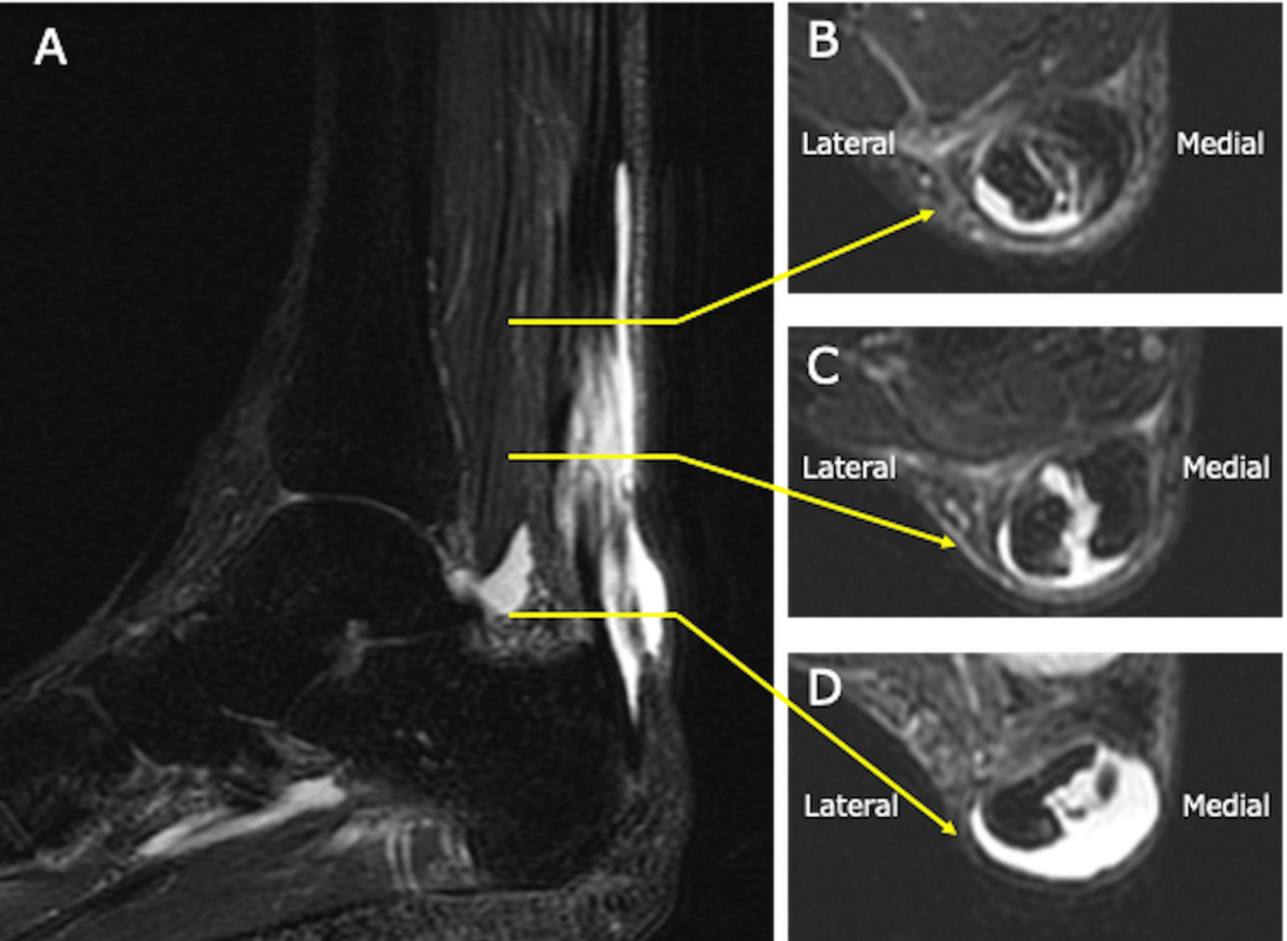
Preoperative magnetic resonance imaging The Achilles tendon showed a split tear involving approximately 50% of the tendon width from the medial side, extending from the proximal portion to the calcaneal insertion, with absence of the medial tendon fibers at the insertion site. A: sagittal view of the Achilles tendon; B: axial view of the proximal Achilles tendon; C: axial view of the mid portion of the Achilles tendon; D: axial view of the proximal Achilles tendon

Plain radiography demonstrated a calcaneal spur at the Achilles tendon insertion and calcific lesions along the course of the tendon (Figure 2). Magnetic resonance imaging revealed a split tear involving approximately 50% of the tendon width from the medial side, extending from the proximal portion to the calcaneal insertion, with absence of the medial tendon fibers at the insertion site (Figure 3). On the basis of these imaging findings, the patient was diagnosed with a partial Achilles tendon rupture following local corticosteroid injection.

Surgical management was selected based on both imaging and clinical findings. Although imaging demonstrated partial continuity of the Achilles tendon, degeneration extended over a wide area. Given the history of corticosteroid injection, advanced qualitative degeneration of the tendon was considered likely. Furthermore, physical examination revealed marked impairment of Achilles tendon function. These factors supported the decision to proceed with operative treatment. Although conventional Achilles tendon reconstruction after resection of the degenerated tendon tissue was initially considered, the patient preferred a minimally invasive strategy. Therefore, Achilles tendon reconstruction using the Midsubstance SpeedBridge technique combined with PRP injection was performed.

**Figure 4:**
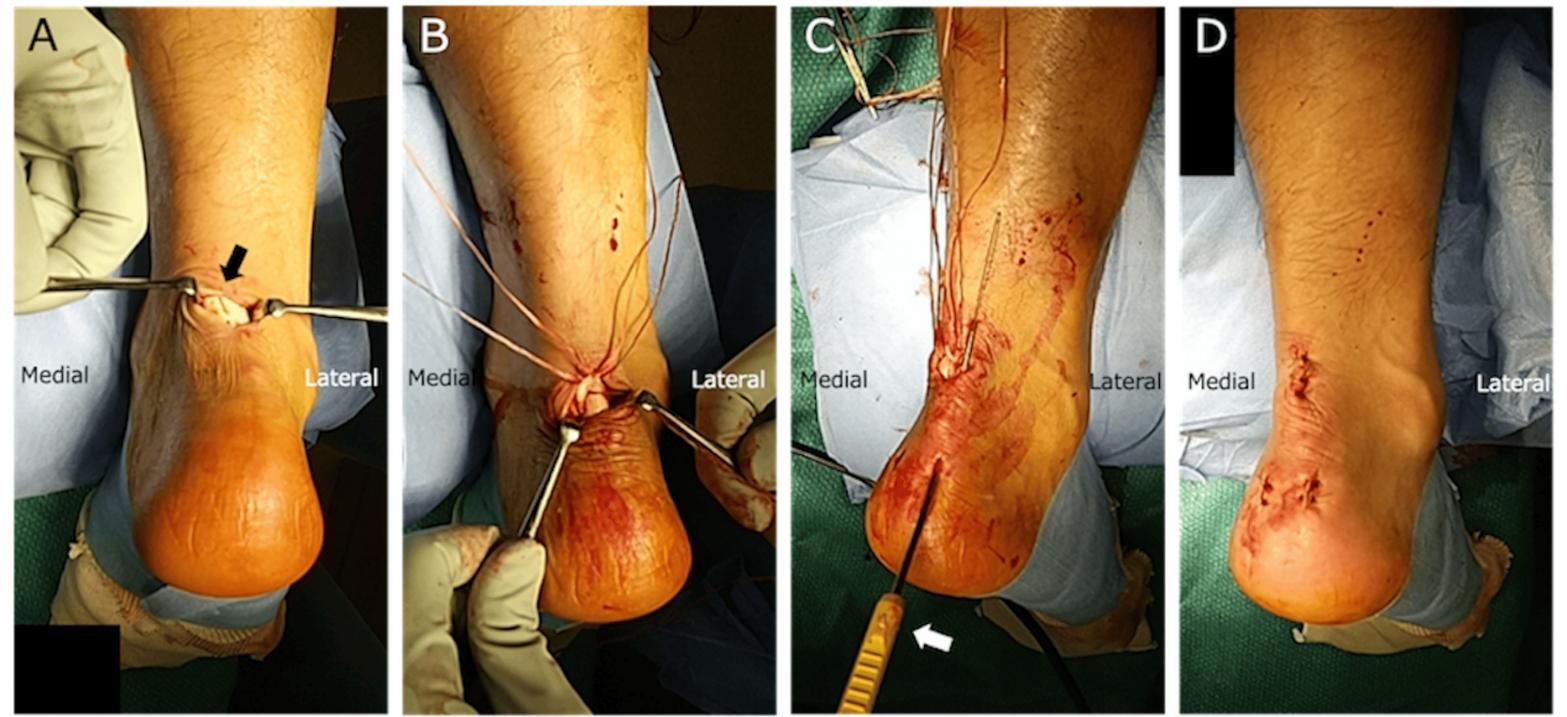
Intraoperative photographs A: the Achilles tendon exhibited scar-like changes and was split in the midline, with more pronounced degeneration on the medial side (arrow); B: one locking suture and two simple sutures were placed in the proximal stump using the Percutaneous Achilles Repair System; C: the proximal suture tapes were passed to the calcaneal insertion using a SutureLasso (arrow) and fixed to the calcaneus with SwiveLock anchors using the Midsubstance SpeedBridge technique; D: surgical wound after closure.

The procedure was performed with the patient in the prone position under combined sciatic and saphenous nerve blocks. A tourniquet was applied to the lower leg, but was not inflated. A 2.5-cm longitudinal skin incision was made approximately 4 cm proximal to the calcaneal insertion of the Achilles tendon. Yellow, clear exudative fluid was observed in the subcutaneous tissue and around the tendon. The Achilles tendon exhibited a scar-like appearance and was split at the midline, with more pronounced degeneration on the medial side (Figure 4A). Using the Percutaneous Achilles Repair System (PARS) (Arthrex, Inc., Naples, Florida, United States), one locking suture and two simple sutures with Suture Tape were placed in the proximal tendon stump (Figure 4B). Using the Midsubstance SpeedBridge Implant System (Arthrex, Inc., Naples, Florida, United States), the proximal Suture Tapes were passed to the calcaneal insertion using a SutureLasso (Arthrex, Inc., Naples, Florida, United States) and fixed to the calcaneus with SwiveLock anchors (Arthrex, Inc., Naples, Florida, United States) (Figure 4C). During fixation, the ankle was positioned in approximately 20° greater plantar flexion than the contralateral side in the prone position, with the ankle allowed to hang freely. After irrigation, the wound was closed, and the procedure was completed (Figure 4D).

**Figure 5:**
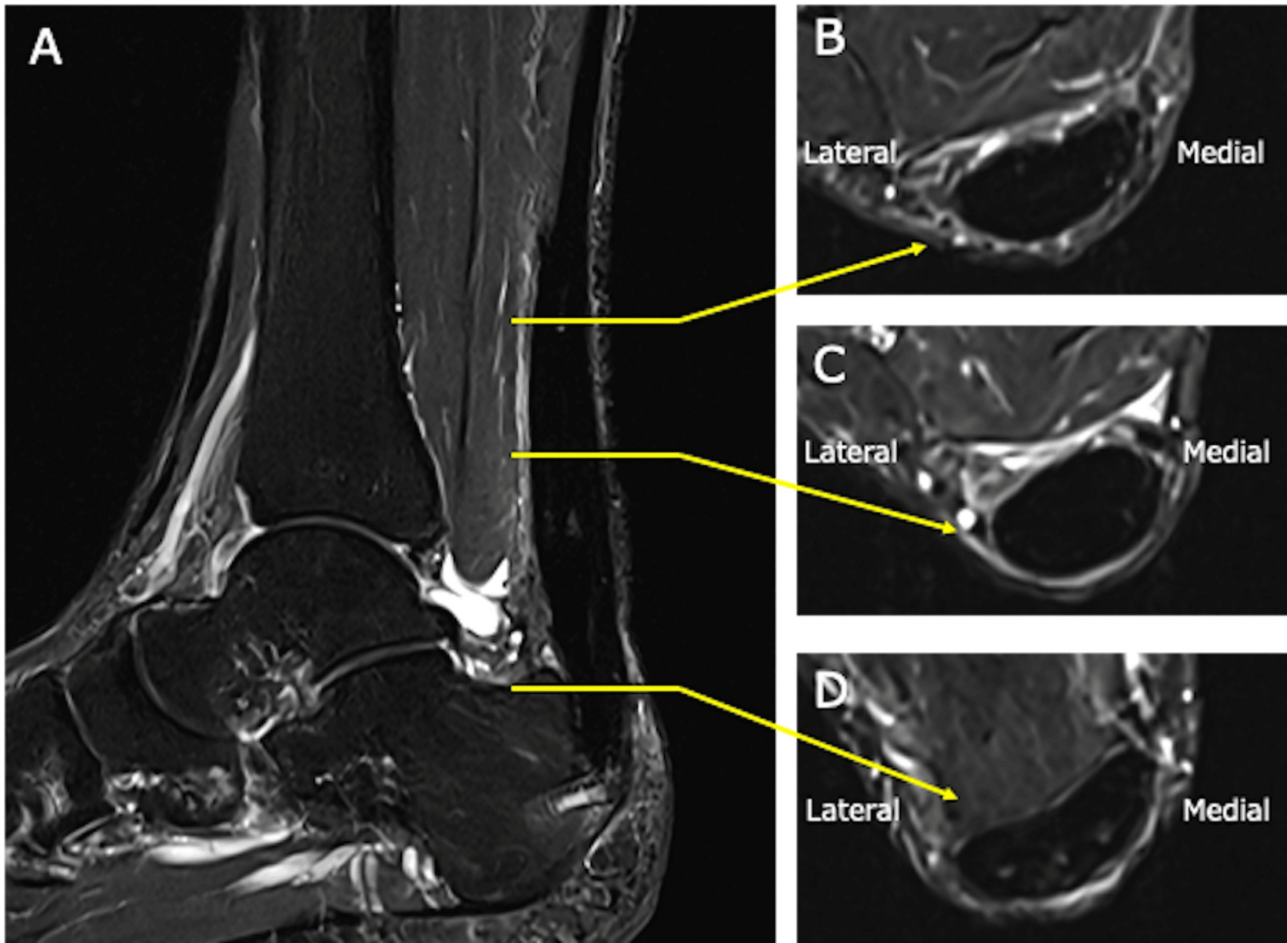
Magnetic resonance imaging at one year postoperatively Favorable healing of the Achilles tendon was observed. A: sagittal view of the Achilles tendon; B: axial view of the proximal Achilles tendon; C: axial view of the mid portion of the Achilles tendon; D: axial view of the proximal Achilles tendon

Postoperatively, the ankle was immobilized in a cast and maintained in a non-weight-bearing condition for two weeks. Ankle range-of-motion exercises were initiated at two weeks after surgery, and full weight-bearing was permitted with a walking boot and heel wedge (Ankle-Foot Orthosis for Achilles Tendon; ADVANFIT Inc., Kumamoto, Japan). The brace was removed at seven weeks postoperatively. After confirmation of the absence of wound infection and wound dehiscence, PRP injection (GPS III) (Zimmer Biomet, Inc., Warsaw, Indiana, United States) was administered at four weeks postoperatively. PRP was injected into the degenerated portion of the Achilles tendon under ultrasound guidance. Bilateral heel raises were initiated at eight weeks postoperatively, followed by single-leg heel raises at 10 weeks. Jogging was initiated at 12 weeks postoperatively after the patient was able to perform single-leg heel raises. The patient returned to golf four months after surgery. At one year postoperatively, no rerupture of the Achilles tendon was observed, and the AOFAS Ankle-Hindfoot scale was 100 points [12]. Magnetic resonance imaging performed at the same time demonstrated satisfactory healing of the Achilles tendon (Figure 5).

## Discussion

The novelty of this report, to our knowledge, is the first description of Achilles tendon reconstruction using the Achilles Midsubstance SpeedBridge technique combined with PRP injection for Achilles tendon rupture following local corticosteroid injection. In previously reported cases, Achilles tendon rupture after local corticosteroid injection has typically been managed with reconstructive procedures, such as tendon transfer and autograft, after debridement of degenerated tendon tissue [2,4]. However, these procedures are invasive and have been associated with a relatively high risk of complications, including delayed wound healing and infection. This case suggests that a minimally invasive strategy combining the Midsubstance SpeedBridge technique with biologic therapy may represent a promising therapeutic option for Achilles tendon rupture following local corticosteroid injection.

The Achilles Midsubstance SpeedBridge technique is a minimally invasive percutaneous repair technique for acute Achilles tendon rupture [13]. A specifically designed jig is introduced into the proximal stump through a small incision, locking sutures are placed using suture tapes, and the suture tapes are guided to the calcaneus and fixed with anchors. This technique has been reported to provide stronger internal fixation than conventional Krackow sutures and to be associated with favorable clinical outcomes [9,10]. In addition, favorable outcomes have also been reported in patients with chronic Achilles tendon rupture treated with the Achilles Midsubstance SpeedBridge technique, indicating that this technique may be applicable to a wide range of clinical conditions [14]. However, to the best of our knowledge, there are no published reports describing the use of the Achilles Midsubstance SpeedBridge technique for Achilles tendon rupture following local corticosteroid injection. In the present case, this technique was applied to reduce tension at the degenerated portion by firmly bridging the proximal healthy tendon to the calcaneus and to prevent rerupture and elongation of the Achilles tendon.

PRP has been shown in basic studies to enhance the healing of injured Achilles tendons through promotion of tenocyte proliferation, increased collagen synthesis, angiogenesis, and modulation of inflammatory responses [15,16]. However, the efficacy of PRP injection as an adjunct to surgical repair for Achilles tendon rupture has not yet been established, and current evidence remains inconclusive [17,18]. Zou et al. reported significantly higher Leppilahti and Short Form-36 (SF-36) scores at 12 months postoperatively in patients who received PRP as an adjunct to surgical repair than in those who underwent surgical repair alone, in a randomized controlled trial of 36 patients with acute Achilles tendon rupture treated surgically using a modified Krackow suture [17]. In contrast, Schepull et al. conducted a randomized controlled trial including 30 patients with acute Achilles tendon rupture treated surgically using the single-loop Kessler technique and reported that, at 12 months postoperatively, no significant difference was observed in the heel raise index between the PRP and control groups, whereas the Achilles Tendon Total Rupture Score was significantly lower in the PRP group, indicating no clinical benefit of adjunctive PRP in that cohort [18]. In the present case, PRP injection was used to promote tendon healing; however, further studies are warranted to determine its clinical benefit. Regarding the timing of PRP administration as an adjunct to surgical repair, previous studies have reported intraoperative PRP injection with or without additional postoperative injections [17-19]. In the present case, PRP injection is not reimbursed under the Japanese National Health Insurance System and, therefore, could not be performed concurrently with the insured surgical procedure. Moreover, to clearly distinguish PRP-related local inflammatory reactions from postoperative infection, PRP was administered four weeks postoperatively after confirming the absence of wound infection. However, intraoperative PRP administration may be more favorable for optimizing Achilles tendon healing.

This case report has several limitations. First, because the present report is based on a single case, the effectiveness and safety of Achilles tendon reconstruction using the Achilles Midsubstance SpeedBridge technique combined with PRP injection cannot be generalized. In addition, it is difficult to clearly distinguish the contribution of PRP injection from the effect of the Midsubstance SpeedBridge technique itself on postoperative outcomes. Second, this case represents a specific clinical condition, namely, partial Achilles tendon rupture following local corticosteroid injection, and it remains unclear whether this technique can be applied to typical chronic Achilles tendon rupture or complete rupture after corticosteroid injection. Third, the patient had a history of hypertension, which has been suggested to be a potential risk factor for structural and degenerative changes in tendons [20]. Hypertension may therefore have contributed to the development of the Achilles tendon rupture in this patient. Fourth, this study did not include objective quantitative strength testing or patient-reported outcome measures, such as the Achilles Tendon Total Rupture Score. Fifth, the follow-up period was limited, and longer-term follow-up is required to evaluate long-term rerupture and functional recovery.

## Conclusions

In this report, we describe a case of partial Achilles tendon rupture following local corticosteroid injection that was successfully treated with minimally invasive Achilles tendon reconstruction using the Achilles Midsubstance SpeedBridge technique combined with PRP injection. This case suggests that a minimally invasive strategy combining the Midsubstance SpeedBridge technique with biologic therapy may be a feasible therapeutic option for Achilles tendon rupture associated with prior local corticosteroid injection. However, because this report describes a single case, the findings cannot be generalized. Further studies with larger case series and longer follow-up are required to clarify the clinical effectiveness of this treatment strategy.
